# Dynamic causal modelling on infant fNIRS data: A validation study on a simultaneously recorded fNIRS-fMRI dataset

**DOI:** 10.1016/j.neuroimage.2018.04.022

**Published:** 2018-07-15

**Authors:** Chiara Bulgarelli, Anna Blasi, Simon Arridge, Samuel Powell, Carina C.J.M. de Klerk, Victoria Southgate, Sabrina Brigadoi, William Penny, Sungho Tak, Antonia Hamilton

**Affiliations:** a Centre for Brain and Cognitive Development, Birkbeck College, University of London, United Kingdom; b Centre for Medical Image Computing, University College London, United Kingdom; c Department of Medical Physics and Biomedical Engineering, University College London, United Kingdom; d Department of Psychology, University of Copenhagen, Denmark; e Department of Developmental Psychology, University of Padova, Italy; f School of Psychology, University of East Anglia, Norwich, United Kingdom; g Bioimaging Research Team, Korea Basic Science Institute, South Korea; h Institute of Cognitive Neuroscience, University College London, United Kingdom

**Keywords:** Effective connectivity, DCM, Infants, Simultaneous fMRI-fNIRS recording

## Abstract

Tracking the connectivity of the developing brain from infancy through childhood is an area of increasing research interest, and fNIRS provides an ideal method for studying the infant brain as it is compact, safe and robust to motion. However, data analysis methods for fNIRS are still underdeveloped compared to those available for fMRI. Dynamic causal modelling (DCM) is an advanced connectivity technique developed for fMRI data, that aims to estimate the coupling between brain regions and how this might be modulated by changes in experimental conditions. DCM has recently been applied to adult fNIRS, but not to infants. The present paper provides a proof-of-principle for the application of this method to infant fNIRS data and a demonstration of the robustness of this method using a simultaneously recorded fMRI-fNIRS single case study, thereby allowing the use of this technique in future infant studies.

fMRI and fNIRS were simultaneously recorded from a 6-month-old sleeping infant, who was presented with auditory stimuli in a block design. Both fMRI and fNIRS data were preprocessed using SPM, and analysed using a general linear model approach. The main challenges that adapting DCM for fNIRS infant data posed included: (i) the import of the structural image of the participant for spatial pre-processing, (ii) the spatial registration of the optodes on the structural image of the infant, (iii) calculation of an accurate 3-layer segmentation of the structural image, (iv) creation of a high-density mesh as well as (v) the estimation of the NIRS optical sensitivity functions. To assess our results, we compared the values obtained for variational Free Energy (F), Bayesian Model Selection (BMS) and Bayesian Model Average (BMA) with the same set of possible models applied to both the fMRI and fNIRS datasets. We found high correspondence in F, BMS, and BMA between fMRI and fNIRS data, therefore showing for the first time high reliability of DCM applied to infant fNIRS data. This work opens new avenues for future research on effective connectivity in infancy by contributing a data analysis pipeline and guidance for applying DCM to infant fNIRS data.

## Highlights

• Connectivity studies give important insights into infant brain development.
• fNIRS is a valuable method for infancy studies, but can we analyse connectivity?
• On fMRI-fNIRS acquired simultaneously, we estimate effective connectivity with DCM.
• We showed high correspondence of DCM values between fMRI and fNIRS data.
• We validated DCM on fNIRS infant data, providing guidance for future projects.

## Introduction

The importance of understanding functional and structural brain connectivity across human development is increasingly being recognized. Whereas the first decades of neuroimaging research examined questions of *functional segregation* and specialisation of brain areas, recent research has begun focusing on understanding *functional integration* and networks in the brain. This approach takes into account how the functional role of each area is largely defined by its connections, and how cognitive functions are usually supported by complex networks and interactions between brain areas (Friston, 1994). Connectivity studies have enriched neuroimaging research with a more comprehensive understanding of how the brain works and develops, playing a fundamental role in revealing neuronal mechanisms behind cognitive processes and psychological domains (Friston, 2011; He and Evans, 2010; Rubinov and Sporns, 2010). The aim of the present paper is to advance our methods for studying connectivity in the infant brain, and develop the use of dynamic causal modelling (DCM) for infant fNIRS data.

Previous studies have used a range of methods to explore connectivity in adults, children and infants. Some researchers have used EEG in awake infants (for some examples see Grieve et al., 2008; Meijer et al., 2016; Orekhova et al., 2014; Righi et al., 2014; Tóth et al., 2017), while most of the studies using fMRI have focused on resting-state connectivity in sleeping infants (Damaraju et al., 2014; Emberson et al., 2015; Fransson et al., 2011; Gao et al., 2016; Kwon et al., 2016; Lin et al., 2008; Lu et al., 2010; Marrus et al., 2017). Although these studies have started to shed light on how connectivity develops over the first years of life, there still is a lot unknown about how different brain areas are functionally related to each other, considering that the brain is constantly changing, maturing over the first years of life (Johnson, 2001; Knickmeyer et al., 2008).

One challenge to the fMRI studies is that connectivity measured during sleep does not display the same patterns of co-activation as in wakefulness, suggesting that sleep stages affect functional networks differently (Tagliazucchi and Laufs, 2014). Moreover, fMRI is very sensitive to movement (Friston et al., 1996). Therefore, head motion is a big limitation for connectivity studies on the developing brain, considering that even very small movements (smaller than 1 mm), that typically occur during natural sleep, will affect functional connectivity estimation. In particular, head movements decrease long-range connectivity and increase short-range connectivity (Deen and Pelphrey, 2012; Power et al., 2012; van Dijk et al., 2012). This means that acquiring usable connectivity data from awake infants and toddlers in the MRI scanner is exceptionally difficult, limiting the range of stimuli and conditions usable with young participants. Therefore, this paper is aimed to validate the use of an advance connectivity technique to allow in the future this method to be used in awake infants.

Recently, functional near-infrared spectroscopy (fNIRS) has emerged as a non-invasive and reliable neuroimaging method, widely used in the developmental field (Ferrari and Quaresima, 2012). In order to detect a hemodynamic response, near-infrared light is projected through the scalp. Skin, bone and human tissues are relatively transparent to light in the near-infrared band of the electromagnetic spectrum, while oxygenated (oxy-Hb) and deoxygenated blood (deoxy-Hb) components present different proprieties of absorption spectra in the near-infrared wavelength region. Differences in the absorption of the oxy-Hb and deoxy-Hb allow us to measure the difference in the haemoglobin concentration. When neurons are activated as a result of functional processing, a localised change in tissue oxygenation occurs in that region. This produces a change in the amount of light absorbed by this tissue and which can be measured by near infrared spectroscopy systems. These measurements are used as surrogates of brain activation (Elwell, 1995; Hoshi, 2016).

There are specific characteristics of fNIRS that make it an ideal neuroimaging technique for exploring the developing human brain (Lloyd-Fox et al., 2010; Wilcox and Biondi, 2015). Firstly, infants have a thinner scalp and less hair compared to adults (aspects that could affect the absorption and the scattering of the light), allowing the near-infrared light to more effectively reach the grey matter. Secondly, the robustness of fNIRS to motion – compared with fMRI - allows us to test infants and toddlers while they are awake and relatively free to move, and so to explore how brain areas differentially respond to cognitive and social tasks. This widens the scope and range of experimental conditions for conducting studies (Holtzer et al., 2011; Solovey et al., 2009). Thirdly, fNIRS is not restricted to a lying down posture, and so is more participant friendly, as infants can sit on or close to their carer throughout the study. Lastly, the portability and the low cost of this equipment has helped increase the use of fNIRS for neuroimaging over the last decade (Hoshi, 2007; Piper et al., 2014). Thanks to these properties, fNIRS use is not only restricted to infants and toddlers, but can also be used in populations with physical or health conditions that may prevent them from being tested in the MRI scanner (Kumar et al., 2017; Leff et al., 2011; Lin et al., 2013; Maidan et al., 2016). Furthermore, the possibility of using fNIRS in more realistic environments including natural movement has facilitated its application in the field of social interaction (Canning and Scheutz, 2013; Holtzer et al., 2011; Pan et al., 2017; Pinti et al., 2015).

Compared to fMRI, fNIRS presents a higher temporal resolution, allowing for rapid data acquisition up to 100 Hz – compared to 1 Hz or less usually provided by fMRI (Kim et al., 1997; Huettel et al., 2004; Weishaupt et al., 2007). This enables fNIRS to provide more time-accurate recordings of the hemodynamic fluctuations of the brain, therefore contributing higher resolution information to studies using connectivity analyses, and those examining the correlations between the time-series and relations between brain areas (Lee et al., 2013).

The increased interest in fNIRS has spurred researchers to develop new methods for data analysis. There are several different options available in the study of brain connectivity in fNIRS and fMRI, and we provide only a brief outline here. The vast majority of fNIRS connectivity studies examine ‘functional connectivity’, defined as *the temporal correlations between spatially remote neurophysiological events* (Friston, 2011). In this context, correlation and coherence methods have been widely applied in neuroimaging, even though they can tell us nothing more than which voxel/channel displays a similar fluctuation pattern to another one that is not necessarily close in space (for reviews see Li et al., 2009; Sun et al., 2004). Functional connectivity is described in terms of ‘statistical dependencies’, so it does not provide any further notion of how experimental condition or psychological variables can mediate its pattern. To link functional connectivity to experimental conditions, it is possible to use a psychophysiological interaction (PPI) model, but to date, only one study applying PPI on fNIRS has been published (Piva et al., 2017). PPI can be estimated using convolution models to test for a possible interaction between a physiological variable and another psychological variable or an experimental factor. Even though PPI is more informative than a basic functional connectivity correlational model, it cannot provide any causality or directionality for the neural dependencies (Friston, 2011). A more informative investigation of connectivity is the study of ‘effective connectivity’, which is related to the *influence that one neuronal system exerts over another* and it infers the relation between hidden neuronal states (Friston, 1994, 2011). One method to explore causality and coupling in brain imaging data uses Granger Causality (GC). Developed in the context of economic science and then applied to neuroscience, this method uses linear regression modelling of stochastic processes to infer causality (Granger, 1969; Seth et al., 2015). However, GC is limited because it bases the inference of causality only on temporal precedence of one time series over another, inferring that earlier responses in a region predicts later responses in another one. This might reflect some imprecision in the estimation of causality if we consider data with low sampling rate (order of seconds) and at the convolution with the HRF process, which usually requires long delays between peaks. Moreover, GC hardly takes into account inter-region variability in the brain and it cannot inform us about the nature of the connections, i.e. whether they are excitatory or inhibitory (Anzellotti et al., 2017; Handwerker et al., 2012). A more precise attempt to define realistic models of human networks is proposed by Dynamic Causal Modelling (DCM), supporting the nonlinear and dynamic nature of interaction between neuronal populations. This method provides a generative model of neuronal and biophysical states underlying specific brain networks, building up a model of the neural patterns in different brain regions and how they interact with each other. DCM is the most accurate technique to estimate effective connectivity, because it can not only evaluate the couplings between brain regions but also how these are influenced by changes in experimental context (Friston, 2011). DCM is actually the first technique that can estimate changes in connectivity not only from endogenous noise, but also from external perturbations. Moreover, this advanced method takes into account two different options in which experimental conditions can enter the model; either through direct influences on specific anatomical brain areas, or through a modulation of the coupling among brain areas, so on the functional connections between regions (Friston et al., 2003). Typically, researchers build a family of models with slightly different connections or experimental contexts, and use Bayesian statistic to determine which model gives the closest description of the data (*inference on model space*) and to estimate the strength and the nature of the connections, excitatory or inhibitory (*inference on parameter space*) (Penny et al., 2004). From this comparison with other connectivity techniques usually used in neuroimaging research, it is appreciably understandable why DCM is so ground-breaking and innovative.

DCM has been developed and widely applied in the context of fMRI (Friston et al., 2011; Schuyler et al., 2010), and it has been adapted for fNIRS on adults (Tak et al., 2015). The aim of the current project was to determine if DCM can be used on infant data as well. In order to validate the use of DCM on infant data, fMRI and fNIRS were simultaneously recorded from a 6-month-old sleeping infant, who was presented with auditory stimuli in a block design. MRI scans of the participant structural images were acquired as well. The application of DCM on infant fNIRS data required several technical challenges to be solved. Firstly, we needed to import the participant structural image in the SPM-NIRS toolbox in order to get a precise spatial registration of the fNIRS optodes on the infant's brain. In order to correctly estimate the light path throughout the brain layers, we then evaluated the NIRS optical sensitivity functions on a high-density mesh based on the segmented structural image of the participant. Finally, the specification of DCM models had to be adapted for infant brain features.

This paper aims to provide solutions to the problems encountered when applying DCM to infant data, and therefore test if this analysis tool, initially developed for adult fNIRS only, can also be applied in the developmental context. The simultaneous fMRI-fNIRS recording allows the validation of this advanced connectivity technique, favouring its application to other fNIRS datasets, obviating the need for MRI. We hope that this project will provide a step toward better studies of functional connectivity with fNIRS, opening doors to new lines of research.

## Material and methods

**Fig. 1:**
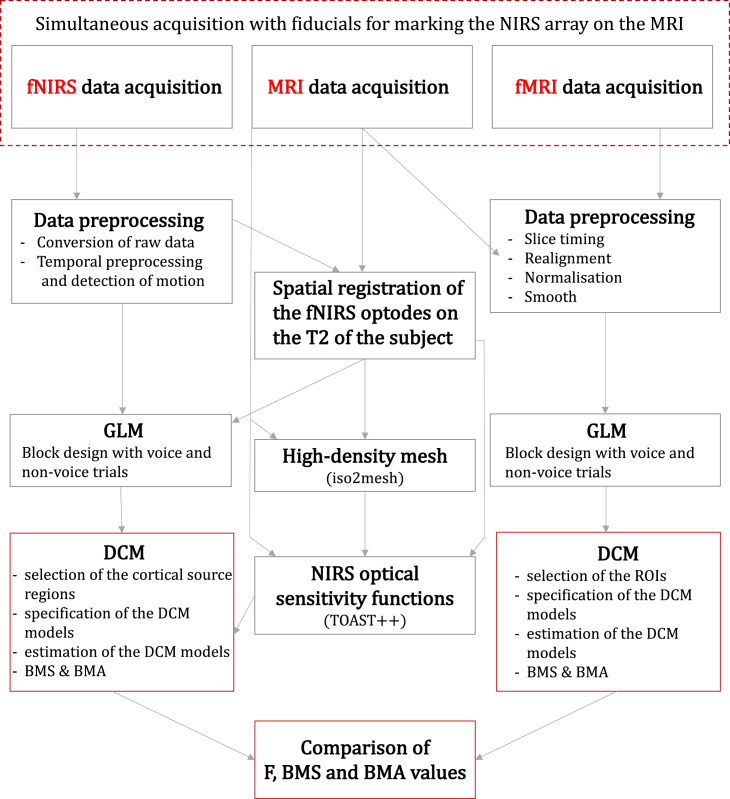
Outline of the analyses conducted in this study for fNIRS, MRI and fMRI.

Fig. 1 displays the outline of the analysis conducted in this study for fNIRS, MRI and fMRI.

### Data acquisition

fMRI and fNIRS data were simultaneously acquired from a single participant (183 days-old) during natural sleep. The infant listened to vocal (e.g. coughing) and environmental (non-vocal) sounds (e.g. running water) interleaved with silence in a protocol previously tested with fNIRS and fMRI separately (Blasi et al., 2011, 2015; Lloyd-Fox et al., 2012a). The stimuli were organized in a block design, with a presentation time of 9 s and a rest period of at least 13 s between conditions. The recording session lasted 11.5 min, and we acquired 24 trials in total, 12 for the voices condition (V) and 12 for the non-voices condition (NV).

#### fMRI

MRI data were acquired using a GE 1.5 T Twinspeed MRI scanner (General Electric, Milwaukee, WI, USA). 320 T2* weighted gradient echo planar multi-slice datasets depicting BOLD (Blood Oxygenation Level Dependent) contrast were acquired in each of 24 non-contiguous near-axial planes (4.0 mm thick with 1.0 mm spacing, 3.5 × 3.5 mm in-plane resolution) parallel to the Anterior Commissure-Posterior Commissure line (TE 57 ms, TR 3000 ms, flip angle 90°, 16:04 min). At the same session, a T2 weighted fast spin echo (FSE) dataset was acquired (256 × 168 rectangular matrix, 2 mm slice thickness, 0 mm slice gap, field of view = 18 cm, TR = 4500, TE = 113 ms, echo train length = 17). Data quality assurance was carried out to ensure high signal to ghost ratio, high signal to noise ratio and excellent temporal stability using an automated quality control procedure. (Simmons et al., 1999). The body coil was used for RF transmission and an 8-channel head coil for RF reception (Simmons et al., 1999). The whole scanning procedure was stopped immediately if the infant awoke and/or expressed discomfort. An experimenter and the parent stood in the scanner room to observe the infant's behaviour at all times.

#### fNIRS

**Fig. 2:**
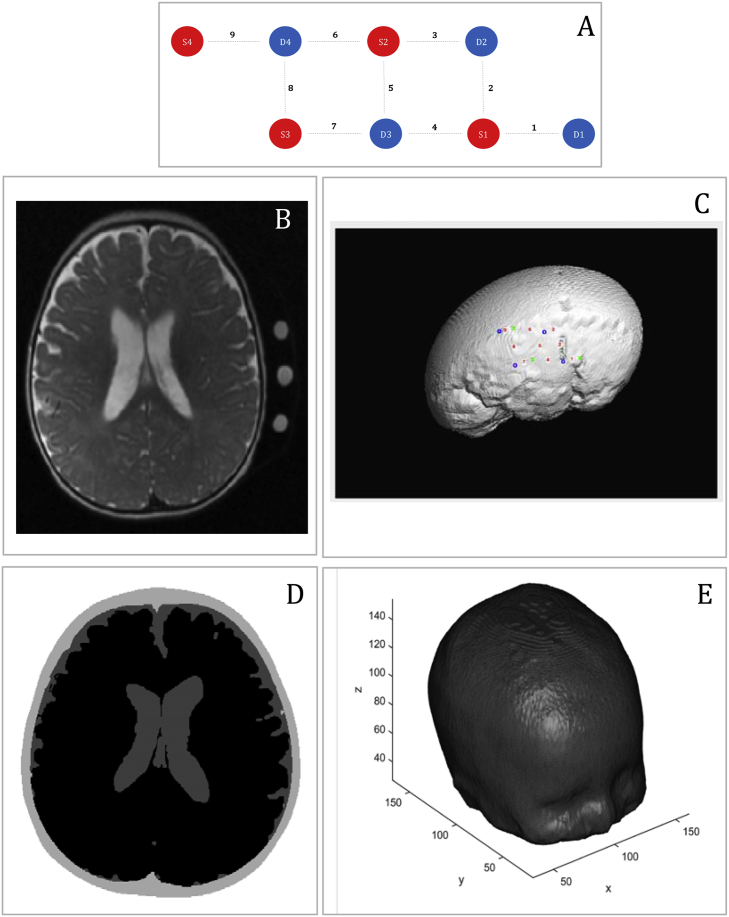
Representation of the main methodological steps. A, Representation of the array. Sources are marked in red, detectors are marked in blue. Channels are marked with grey dotted lines and the channel number is indicated. B, T2 structural image of the participant with fiducials for the fNIRS channels. C, Output from the NIRS-SPM toolbox, spatial registration of the optodes over the T2 of the infant imported with the NFRI toolbox. Green ‘x’ are the sources, blue ‘o’ are the detectors and channels are labelled with red numbers. D, 3-layers segmentation from the T2 structural image of the infant. E, High-density volumetric tetrahedral mesh created with *iso2mesh* toolbox from the segmented structural image of the infant.

The fNIRS array (UCL Optical Imaging System (Everdell et al., 2005)) was placed over the right temporal lobe, and included 9 source-detector pairs (channels), defined by 4 sources and 4 detectors, with a 2 cm source-detector separation. The sources in the NIRS system provided light at 770 nm and 850 nm, and the sampling rate of acquisition was 10 Hz. MRI fiducial markers (vitamin E caplets) were attached to the inter-optode spaces of the NIRS array to guide the co-registration of the NIRS data onto the MRI image. Fig. 2A shows a design of the array.

Our brain activations and connectivity analyses were restricted to the right hemisphere, because the fNIRS array was placed over the right hemisphere only, in order to optimise the number of sources and detectors available from the system, the difficulty of fitting the NIRS headgear within the restricted space in the MRI coil, and also considering the wide literature that suggests the main role of the right hemisphere in social auditory processing (Belin et al., 2000; Blasi et al., 2011; Grossmann et al., 2010; Lloyd-Fox et al., 2012a).

### Data pre-processing

#### fMRI

All fMRI images were analysed using Statistical Parametric Mapping software (SPM12) (Friston and Ashburner, 1994). Scans were corrected for slice timing and realigned for head movements. Images were then normalized to the T2 image of the participant, and smoothed using a 7-mm full-width at half-maximum isotropic Gaussian kernel.

#### fNIRS

The fNIRS data were analysed using the SPM-fNIRS toolbox, a SPM12 based software for statistical analysis of fNIRS signal (Tak et al., 2016; Ye et al., 2009). The raw intensity data were converted to haemoglobin changes using the modified Beer-Lambert Law (differential path length factor, DPF = 5.13 (Duncan et al., 1995)). The spatial pre-processing registering the fNIRS channels over the native space of the participant was performed (see specific paragraph below). The temporal pre-processing included the removal of physiological noise (5th order Butterworth high-pass filter: 0.008 Hz; band-stop filter: 0.06-0.16 – 0.8-1.8 Hz), and the reduction of motion artefacts with spline interpolation. Artefacts were detected as changes in moving standard deviation larger than 3 μM using a 1 s sliding window (Scholkmann et al., 2010). Our analysis of fNIRS data is based on changes in oxy-Hb, following the only study to date that has applied DCM to fNIRS data (Tak et al., 2015). Additionally, previous fNIRS studies with infants typically do not find any statistically significant deoxy-Hb changes (for some examples see: Grossmann et al., 2013; Lloyd-Fox et al., 2010; Lloyd-Fox et al., 2015; Southgate et al., 2014).

##### Spatial registration

A precise estimation of optode positions and reference points is crucial for calculating connectivity, therefore, particular attention needs to be dedicated to the fNIRS spatial registration to the MRI images. Location of the reference points and of the optode positions are required as an input of the fNIRS spatial processing to calculate the brain area covered by the NIRS channels. One option would be to use readily available adult MRI templates. However, this is not an ideal solution as the infant brain is not a reduced-size version of an adult brain (Sanchez et al., 2012b). Instead, we used the participant's own structural image for spatial co-registration of the NIRS data.

Optode positions on the head were manually estimated from the fiducial markers on the T2 structural image of the participant. In particular, we defined projection points from each fiducial onto the scalp as the location of each NIRS channel (Fig. 2B). We estimated the centre of the fiducial as the middle point between each source and detector, therefore they mark the centre of the channel. From here, we calculated the exact coordinates of each optodes on the infant's head.

The structural image of the infant was imported in the SPM-fNIRS toolbox, using custom modified code from the NFRI toolbox (Okamoto et al., 2004). On this structural image, the reference points and the optodes were plotted (see Fig. 2C).

### Segmentation and creation of the mesh

Two structural scans were recorded from the participant: one immediately before and another one immediately after functional data collection. Superimposition of both images revealed that the infant had barely moved between the two time points, therefore the two images were averaged and upsampled to improve their quality (Manjn et al., 2010). After that, the image was flattened to remove intensity level inhomogeneities caused by the magnetic field, and then, using in house scripts written in Matlab, its background was removed. The structural scan was skull stripped with FSL (http://fsl.fmrib.ox.ac.uk/fsl/fslwiki/FSL) using the BET (Brain Extraction Tool) routine including the functions BET2 (to isolate the brain) and BETSURF (to separate the scalp and inner skull surfaces) (Jenkinson et al., 2012). The brain image was then further processed with SPM's SEGMENT option, using tissue probability maps from an age appropriate segmented template (from the Neurodevelopmental MRI Database, of the Univ. of South Carolina http://jerlab.psych.sc.edu/NeurodevelopmentalMRIDatabase/), very light bias regularisation (0.0001), and FWHM Gaussian smoothness of bias with 30 mm cut-off. At the end of this process, each infant's structural scan was segmented in 3 layers: skin and skull and extra-cerebral tissue, CSF, and brain (grey plus white matter). Using another set of Matlab scripts, the 3 layers were post-processed to fill in gaps and ensure that all voxels were assigned the correct labels. As explained in the previous section, the segmented images were then used to provide the necessary anatomical information to the fNIRS data reconstruction step (see Fig. 2D).

From the 3-layers segmentation image, a high-density volumetric tetrahedral mesh was created using *iso2mesh* toolbox (Fang and Boas, 2009) (see Fig. 2E). Using in-house custom code, the optodes coordinates were converted from the MRI structural image context to mesh-based context. (For the estimation of the optode positions, the reader is referred back to the section on *Spatial registration* paragraph). The optode locations on the mesh and the mesh itself created from the structural scan were used as inputs for the estimation of the NIRS optical sensitivity functions.

### NIRS optical sensitivity functions

Application of the DCM technique requires estimates of the sensitivity of the optical measurements at different wavelengths to changes in the chromophore concentrations of interest. In the context of diffuse optical imaging, these sensitivity functions are referred to as photon measurement density functions (Arridge and Schweiger, 1995; Arridge, 1995). The requisite sensitivity functions are calculated from products of the forward field generated by a given optical source, and the adjoint field generated by placing an equivalent optical source at the location of a detector. To compute the forward and adjoint fields the diffusion equation was employed with a Robin boundary condition

$$\left(\nabla \cdot \text{κ}\nabla +{\text{μ}}_{\text{a}}\right)\phi \left(\text{r}\right)=0\;\left(\text{r}\in \text{Ω}\right),$$

ϕ(r)+2Aκn⋅∇ϕ(r)=q(r∈∂Ω),

where ‘r’ is a point in space, Ω is the computational domain with boundary ∂Ω,ϕ is the fluence rate resulting from application of the physical or adjoint source q, $\text{κ}={\left(3\left({\text{μ}}_{\text{a}}+{\text{μ}}_{\text{s}}\right)\right)}^{-1}$ is the diffusion coefficient, ${\text{μ}}_{\text{a}}$ and ${\text{μ}}_{\text{s}}$ are the wavelength dependent baseline absorption and scattering coefficients, A is a term accounting for the index of refraction mismatch at the boundary, and n is the outward normal to the boundary (S R Arridge et al., 1993).

The TOAST++ toolbox was used to solve the diffusion approximation numerically via the Finite Element Method (Schweiger and Arridge, 2014). In each case the properties of the source and detector were specified according to physical measurements, and the wavelength dependent baseline absorption and scattering coefficients were derived from a previous study performed on neonates (Singh et al., 2014).

### General linear model for fMRI and fNIRS

For both fMRI and fNIRS data, the evoked hemodynamic responses were modelled as a delta function convolved with a hemodynamic response and its spatial and temporal derivatives within the context of the General Linear Model (GLM). Onsets of voice (V) and non-voice (NV) trials were specified in seconds.

### Selection of ROIs/cortical source regions and definition of the DCM models

**Fig. 3:**
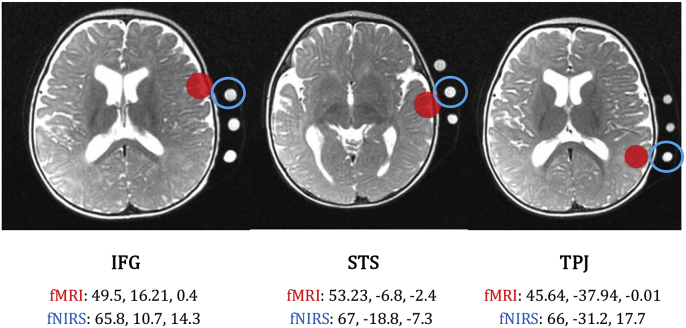
Coordinates and plots of fMRI ROIs and fNIRS cortical source regions on the T2 structural image. IFG, inferior frontal gyrus; STS, superior temporal sulcus; TPJ, temporo-parietal junction. The red sphere corresponds to the fMRI ROIs and the blue circle to the fNIRS cortical source regions.

In order to estimate effective connectivity with DCM, we selected a priori volumes of interest in inferior frontal gyrus (IFG), superior temporal sulcus (STS) and temporo-parietal junction (TPJ). The selection of the fMRI ROIs was based on maximum activation peaks showed from GLMs and considering previous literature on auditory processing (Belin et al., 2000; Blasi et al., 2011; Grossmann et al., 2010; Lloyd-Fox et al., 2012a,b). The fNIRS cortical source regions were defined based on previous coregistration works (Lloyd-Fox et al., 2014), considering the coordinates of the closest channel to the region of interest (fNIRS source regions need to be specified on the participant's cortical surface). Both in the fMRI and in the fNIRS contexts, we extracted principal eigenvariates in 4 mm spheres centred in ROIs/cortical source regions. See the specific coordinates for both fMRI and fNIRS in Fig. 3.

Our DCM analyses were restricted to the right hemisphere, to the volume covered by our fNIRS array layout. We modelled the differential state equations on different seed regions of interest (IFG, STS, TPJ in the right hemisphere), with fMRI and fNIRS. Each DCM model was defined by (i) a set of *intrinsic connections* (A) that specify the present state of one neuronal population, (ii) a set of *modulatory connections* (B) that indicate which intrinsic connections are dependent on experimental manipulations, (iii) *driving inputs* (C), considered as direct influences of the stimuli on the neural activity of involved regions of input connections (Friston et al., 2003).

**Fig. 4:**
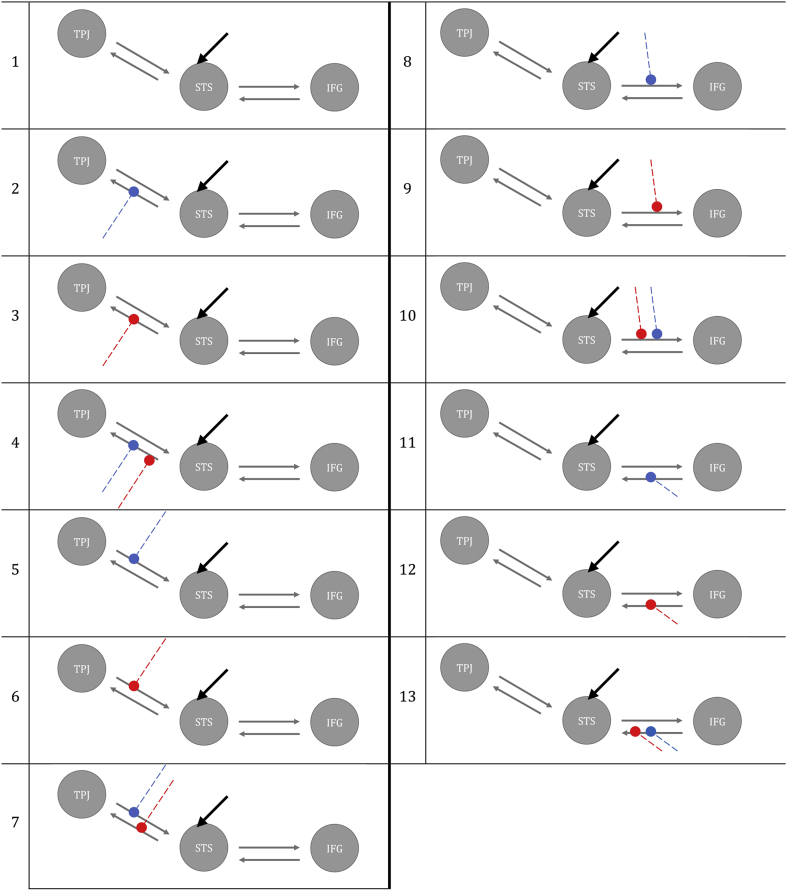
Models representation of our 13 hypotheses. Grey arrows represent the fixed intrinsic connections between the IFG, STS and TPJ; black arrows pointing at STS represent the input; blue lines represent the modulatory effect of NV and red lines the modulatory effect of V.

Thirteen alternative models with different modulatory effects of V and NV were constructed with DCM-SPM toolbox for the fMRI data (Friston et al., 2003) and with DCM-fNIRS toolbox for the fNIRS data (Tak et al., 2015). All models were defined as bilinear and deterministic. Auditory input for both V and NV entered the network by directly activating STS across all models. In all the hypothesized models, we fixed bidirectional intrinsic connection between STS and TPJ and STS and IFG. The models varied for the presence or absence of modulatory effects of auditory processing of V and NV on the connections. See all the possible models in Fig. 4.

### Specification and estimation of the DCM models

The DCM models were fitted to the optical density signal averaged across trials. Specifically, the generative model of fNIRS data was created by linking the optics equation to the hemodynamic and neurodynamic equations (Arridge, 1999; Buxton et al., 2004; Cui et al., 2010; Delpy et al., 1988; Friston et al., 2003). The DCM parameters were then estimated from fNIRS data using an established Bayesian framework (variational Laplace), which enabled inference about changes in directed connectivity at the neuronal level (Friston et al., 2007; Penny, 2012). In this study, we augmented the optics model used for DCM-fNIRS analysis (Tak et al., 2015) by adding a scaling factor to a sensitivity matrix:

$$\left[\begin{array}{c} y\left(\lambda_{1}\right)\\ y\left(\lambda_{2}\right) \end{array}\right]=\left[\begin{array}{cc} \varepsilon_{H}\left(\lambda_{1}\right)W_{H}S\left(\lambda_{1}\right) & \varepsilon_{Q}\left(\lambda_{1}\right)W_{Q}S\left(\lambda_{1}\right)\\ \varepsilon_{H}\left(\lambda_{2}\right)W_{H}S\left(\lambda_{2}\right) & \varepsilon_{Q}\left(\lambda_{2}\right)W_{Q}S\left(\lambda_{2}\right) \end{array}\right]\left[\begin{array}{c} \Delta H_{c}\\ \Delta Q_{c} \end{array}\right]$$

where y is measurements of optical density changes; $\varepsilon_{H}$ and $\varepsilon_{Q}$ are extinction coefficients for oxy-Hb and deoxy-Hb; $W_{H}$ and $W_{Q}$ are factors for correcting pial vein contamination of fNIRS measurements; $\Delta H_{c}$ and $\Delta Q_{c}$ are oxy-Hb and deoxy-Hb in the cortical source regions of interest; and $S=k\cdot S_{0}$ where $S_{0}$ is the sensitivity function calculated from products of the forward field and the adjoint field, and *k* is a scaling term. We treated this scaling term *k* as free parameters with informed priors, to accommodate a variation in source strength (and detection efficiency). This enabled us to calculate a matrix of the sensitivity, *S*, to the absorption coefficient changes, using outputs of the Toast software (Schweiger and Arridge, 2014).

### fMRI-fNIRS DCM model comparisons

After the statistical estimation of each model for both fMRI and fNIRS data, comparisons of the DCM models estimated with fNIRS and fMRI were performed to evaluate effective connectivity correspondence between the two methodologies. The comparison of the DCM models was mainly based on the variational Free Energy (F), which is thought to have the best model selection ability and is highly recommended for comparisons, mostly in high signal-to-noise ratio conditions, as with infant data (Penny, 2012). Bayesian model selection (BMS) was applied in order to estimate the best model on the fMRI and fNIRS data (Friston et al., 2016; Stephan et al., 2007). Our aim was not to investigate why a specific model wins in the BMS comparison, but to see whether there is any convergence between fMRI and fNIRS data, thus answering a methodological rather than a cognitive question. We then estimated the strength of connections for each model (Bayesian Model Average, BMA) for both fMRI and fNIRS to investigate whether there is any correspondence between the two methodologies.

## Results

### Activation results

Prior to DCM analyses, we explored brain regions activated by the two experimental conditions with fMRI and fNIRS.

As previously shown, IFG, STS, and TPJ were involved in the auditory processing in both fMRI and fNIRS (Blasi et al., 2011, 2015; Lloyd-Fox et al., 2012a). The detailed comparison between fMRI and fNIRS activations simultaneously recorded is object of another study in preparation (A. Blasi, B. Manini, S. Brigadoi, R. Cooper, G. Barker, S. Wastling, Lloyd-Fox, M.H. Johnson and C.E. Elwell, Simultaneous fMRI and fNIRS analysis in young infants, Poster presentation at 2016 Biennial Meeting of the Society of functional near-infrared spectroscopy, Paris).

### DCM results

**Fig. 5:**
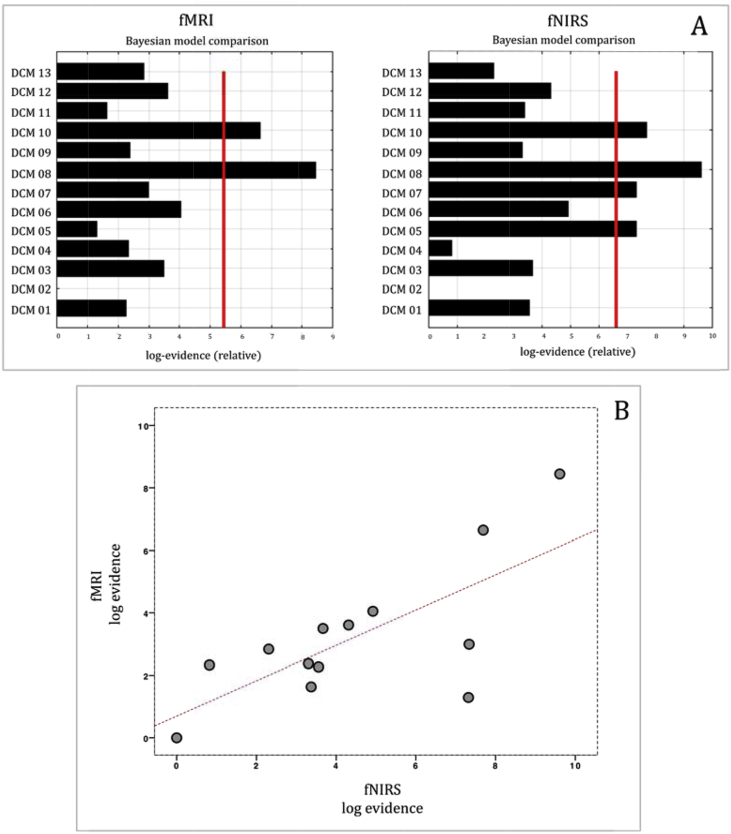
A, Bayesian Model Selection estimated with fMRI and fNIRS. B, Pearson correlation plot between fMRI and fNIRS log evidence of the 13 DCM models.

**Fig. 6:**
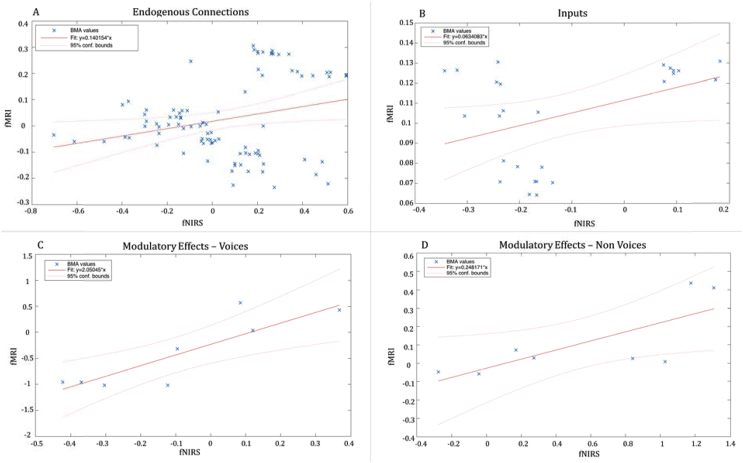
Robust regressions between BMA values estimated with fMRI and fNIRS. A, Scatterplot of the robust regression on the 91 endogenous connections BMA values (4 fixed connections + 3 connections within the area for each model). B, Scatterplot of the robust regression on the 26 inputs (one input for each model for the two conditions). C, Scatterplot of the robust regression on the 8 modulatory effects on V. D, Scatterplot of the robust regression on the 8 modulatory effects on NV.

Fig. 5 shows the correspondence of F values and the BMS comparison between fMRI and fNIRS. Fig. 6 shows the correlation between BMA values estimated with fMRI and fNIRS.

We found a high correspondence between fMRI and fNIRS DCM models (Fig. 5A). Moreover, BMS showed model 8 as the best model for both fMRI and fNIRS, which presented modulatory effect of NV in the STS→IFG connection. Pearson correlation confirmed a strong relationship between fMRI and fNIRS BMS log evidence (r = 0.718, p = 0.006) (Fig. 5B).

To assess the strength of the correspondence between the fMRI-DCM and the fNIRS-DCM results, we used a robust-regression method to compare the parameters estimates obtained for all of the models across the two datasets. We find that the strength of the connections (BMA values) estimated for the 13 models with the two methodologies are highly related for the endogenous connections (Fig. 6A) (F(1,89) = 5.55, p = 0.020, R^2^ = 0.058), the inputs (Fig. 6B) (F(1,24) = 4.35, p = 0.047, R^2^ = 0.153), and the modulatory effects (Fig. 6C and D) on V (F(1,6) = 16.4, p = 0.006, R^2^ = 0.732) and on NV (F(1,6) = 6.65, p = 0.041, R^2^ = 0.526).

**Fig. 7:**
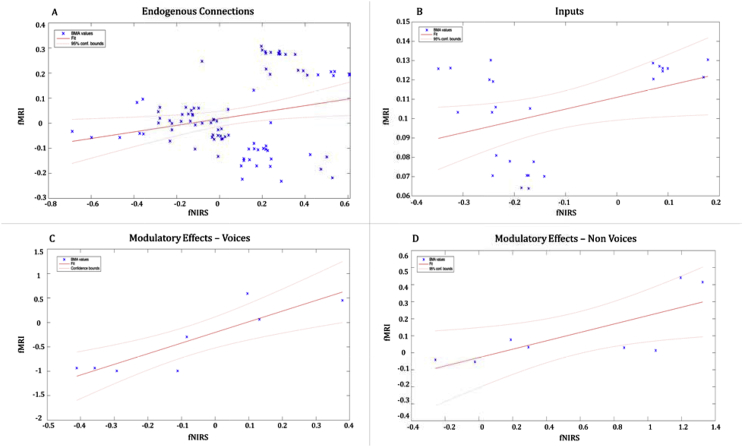
Regressions with bootstrap method between BMA values estimated with fMRI and fNIRS. A, Scatterplot of the robust regression on the 91 endogenous connections BMA values (4 fixed connections + 3 connections within the area for each model). B, Scatterplot of the robust regression on the 26 inputs (one input for each model for the two conditions). C, Scatterplot of the robust regression on the 8 modulatory effects on V. D, Scatterplot of the robust regression on the 8 modulatory effects on NV.

In addition, we have repeated the regression analysis with a bootstrap method for the endogenous connections (Fig. 7A) (F(1,89) = 5.63, p = 0.019, R2 = 0.059, C.I. = 0.114, 0.855), the Inputs (Fig. 7B) (F(1,24) = 4.67, p = 0.040, R2 = 0.163, C.I. = −1.712, −0.353), the modulatory effects (Fig. 7C and D) on V (F(1,6) = 20.2, p = 0.004, R2 = 0.771, C.I = 0.17, 0.43) and on NV (F(1,6) = 7.6, p = 0.033, R2 = 0.559, C.I = 2.67, 3.7).

## Discussion

Developing and applying appropriate analysis methods for the study of neural connectivity in the infant brain is a complex technical challenge. Here we provide a proof-of-principle for the application of DCM to infant fNIRS data. We took a unique dataset, comprising a simultaneous fMRI-fNIRS recording on a single infant listening to different sounds during sleep. We applied the same DCM analyses to both fMRI and fNIRS data, overcoming a number of challenges in the development of the fNIRS analysis stream. We find converging results between both the fMRI and fNIRS methods, and thus our paper demonstrates both the feasibility and validity of applying DCM to infant fNIRS data. We discuss our results first in terms of the broader field of infant connectivity measures and then in terms of future directions. There are major limitations in using fMRI to investigate connectivity in development and this paper provides a solution by showing that reliable results can be obtained with fNIRS instead.

There are a number of different ways in which neuroimaging data (specifically fMRI or fNIRS) can be used to estimate the connectivity between different brain regions (for a review see Li et al., 2009), but only DCM provides detailed information of the relation and the causality between brain areas (Friston, 1994). In particular, we want to point out that most of the other connectivity methods just ask ‘does signal A correlate with signal B’ without enquiring about where the signals come from. In contrast, DCM builds a full model of the neural origins of the signals and models connectivity at the neural level. For this reason, DCM requires much more detailed information about the origin of the signals, compared to PPI.

To convert the scalp-level fNIRS concentrations into estimates of neural activity, we need to know the precise anatomy of the head and its optical properties. Hence, applying fNIRS-DCM to a new (non-adult) population requires high-quality anatomical images, very clean segmentations and accurate estimation of the NIRS optical sensitivity functions. Here we were able to use the infant's high-resolution anatomical images to complete this step. However, future studies could use pre-segmented infant templates, whenever these are available (Richards et al., 2016; Sanchez et al., 2012a,b). Note that despite the low spatial resolution of fNIRS, it is not possible to perform DCM-fNIRS without any detailed anatomical data, because DCM-fNIRS relies on a sophisticated model of how neural activation is filtered via the haemodynamic response and the optical properties of the head to give the fNIRS signal recorded at the scalp. This calculation requires a high-resolution anatomical input, whether from each individual participant or from an age-appropriate template.

Several technical issues had to be overcome in order to apply DCM on infant data. Firstly, we successfully imported the T2 of the participant in the NIRS toolbox in order to obtain an accurate estimation of the connectivity results. We were able to estimate the NIRS optical sensitivity functions, which required participant T2 segmentation, the creation of a high-density mesh and estimation of the optodes location. All these technical challenges have been solved and specific features have been adapted for infant data, allowing other researchers to use DCM on fNIRS data without the need for MRI.

Although our results refer to a single case study, this study shows that DCM for fNIRS is valid for exploring effective connectivity in infant data. We hope that this project can open the door to a new line of research where fNIRS can provide useful information about effective connectivity in infants and toddlers.

### Advice for future users and limitations of this work

Starting from this validation work of DCM in infants, we provide here some advice for future studies, so that other researchers can take this paper as reference and guidance for the use of DCM with infant fNIRS data. As described above, an anatomical MRI scan of the participant is not essential if high-resolution age-appropriate templates are available. We recommend the use of the ‘*Neurodevelopmental MRI Database*’, which is the most accurate MRI database available so far in the developmental neuroscience field, both for quality of images and precision of the age range (Richards et al., 2016; Sanchez et al., 2012a, 2012b). The NIRS optical sensitivity functions can be estimated on the same age-range specific structural template as is used for the spatial registration, instead of using the T2 scan of the participant as we did. It is worth reminding the reader that in this specific study, we performed the spatial registration and the NIRS optical function estimation on the structural scan of the participant, not on a template of the appropriate age as we have suggested for future users. However, while it is established for adults that template-based methods work more precisely than registration performed on a single structural scan, there is no evidence that is still the case in the developmental field. Considering that we have not performed the same processing and connectivity estimation also on an age-range specific structural template, we cannot infer whether connectivity results could benefit from the choice of one or another method.

Additionally, accurate location of the optodes is required in fNIRS data analysis. Most of the researchers use pictures of the fNIRS hat on the participant's head to mark reference points and optodes location on an MRI template, even though this method might result in location inaccuracies, due for example to warping of the pictures - which might lead to erroneous estimation of the distance between points - or human mistakes in positioning the marks on the MRI template. Alternatively, researchers can register optode locations with a digitizer, such as Polhemus Digitising System (http://polhemus.com/scanning-digitizing/digitizing-products/), with the possibility to take into account infants' movement during the recording. However, we acknowledge that the use of a digitizer is not always possible and realistic with restless and fidgety infants, so the support of pictures for spatial registration is still currently used in the developmental research practice. With both these methods mentioned, optode locations and reference points can then be plotted on an MRI template, and then used as input for the spatial processing in both SPM-NIRS and *dcm_fnirs* toolboxes. The use of an age-specific template for both spatial registration and for the estimation of the NIRS optical sensitivity functions will allow researchers to apply DCM to every infant dataset and follow our pipeline, avoiding the need for the acquisition of the MRI for each participant tested.

Researchers working with infants and interested in network analysis should bear in mind that motion artefacts are recognized as one of the major methodological challenge for functional connectivity studies (Satterthwaite et al., 2017). In order to avoid false positive, data need to be as clean as possible, which is not always the case of awake and behaving infants, even after an appropriate pre-processing. However, giving that the present validation of DCM has been performed on an asleep participant, we cannot advise future researchers in how the method deals with noisy data. The avoidance of false positive in connectivity analysis in highly dependent on the pre-processing steps performed before estimating connectivity, and future studies focusing on the application of several pre-processing streams and cleaning methods might elucidate how accurate is DCM with noisy infant data.

### Future directions

In the near future, we aim to apply DCM on a full infant dataset, in order to estimate effective connectivity without relying on the support of MRI scans. While this present study is focused on the application and validation of the method, we hope to be able to further utilise DCM in infants in order to ask psychological and cognitive questions. DCM-fNIRS is already available in the SPM12 software and we will be involved in integrating the scripts we modified for the specification of the DCM models in the main *dcm_fnirs* package, in order to facilitate and promote the application of this technique by other research teams.

We hope that this study can aid developmental neuroscientists who are interested in exploring brain connectivity in infancy and early childhood. We encourage the study of effective connectivity using DCM in the developing brain, in order to gain a deeper understating of brain mechanisms during specific conditions, for example social interaction. We are confident that this study will be a good reference for a new line of research that will not only shed light on how connectivity develops and changes during specific stimulation, but provides researchers who use fNIRS with a valuable tool to interpret their data and to draw psychological conclusions.

## Conflicts of interest

None.
